# Exilic (Art) Narratives of Queer Refugees Challenging Dominant Hegemonies

**DOI:** 10.3389/fsoc.2021.641630

**Published:** 2021-05-10

**Authors:** Fabian Holle, Maria Charlotte Rast, Halleh Ghorashi

**Affiliations:** Department of Sociology, Vrije Universiteit Amsterdam, Amsterdam, Netherlands

**Keywords:** queer, refugees, art practices, agency, paradox, liminality, hegemony, reflection

## Abstract

Within the Dutch hegemonic discourse, the “migrant other” is portrayed as almost incompatible with “national culture” while it is simultaneously pressured to assimilate. This creates paradoxes for the queer refugee participants in this study. When these refugees assimilate, they risk reinforcing the dominant discourse considering their group as the “backward other”. When they do not assimilate, they are considered not “properly” Dutch. This paper explores how queer refugee artists can unsettle such dominant exclusionary discourses through exilic (art) narratives. Their experiences of exilic positioning (being neither there nor here) and queer liminality (e.g., nonbinary gender identifications) and their intersectional positionalities situate these artists in various “states of in-betweenness”. Although these states may be challenging, this paper shows how they can also stimulate agency. Inspired by a feminist approach, this study aimed to co-create knowledge *with* rather than *about* participants, focusing on creativity and resilience. Methods included biographical interviews and an arts-informed component in which participants were invited to create artistic works concerning their experiences during COVID-19 for an online platform. This study shows how the research participants challenge hegemonic discourses at various levels, using multiple modes of reflection and creation while engaging with their in-between situatedness. At the individual level, they challenge discourses by exploring (or performing) their non-conforming queer positioning through their art practices. At the communal level, plural reflexivity is triggered via art shared within and outside the community. At the societal level, queer refugees exercise activism creatively through images, songs or performances.

## Introduction

Around the world, there are lesbian, gay, bisexual, transgender and queer (LGBTQ+) individuals who are forced to flee their countries. This may be due to either their sexual orientation or gender identity (SOGI) or contextual circumstances such as war. Queer refugees often report a range of traumatic events from their origin countries, including physical, emotional, and sexual assault; work and housing discrimination; forced prostitution; and forced heterosexual marriage (Shidlo and Ahola, 2013).

Queer refugees then face additional challenges in their host countries. They often must contend with the possible continuation of harassment by their ethnic community due to their SOGI (Hopkinson et al., 2017). Moreover, this group of migrants often does not access social support from the wider host LGBTQ+ community due to feelings of shame and trauma resulting from experiences of violence, as well as cultural differences (Shidlo and Ahola, 2013). Consequently, queer refugees are particularly vulnerable to exclusion and subsequent isolation (Elferink and Emmen, 2017). The inability to talk openly about experiences before and after arrival in the host country may increase anxiety, insomnia, stress and depression (Elferink and Emmen, 2017).

While some studies focus on accumulating the challenges faced by queer refugees, others show that victimizing refugees is problematic because it reproduces social hierarchies and ways of “othering” (Ghorashi, 2018). In other words, victimizing, or focusing on vulnerabilities, reproduces and reinforces hegemonic perceptions of refugees as, for instance, “dependent,” “unimaginative,” “deviant” and “deficient”. The current study is situated within the Netherlands, which is often considered a progressive and tolerant country. However, in Dutch hegemonic discourses, migrants, and particularly Muslims, are often portrayed as incompatible with Dutch culture (Mepschen et al., 2010). Due to the Netherlands' (assumed) pioneering role regarding women's and gay emancipation, a dominant and nostalgic perception developed that prior to the arrival of Muslims, women's and gay emancipation was almost complete (Wekker, 2009). Islam is therefore often portrayed as a threat to these emancipatory developments due to its alleged incompatibility with “emancipated liberal values” (Mepschen et al., 2010). This has partly contributed to a shift within the Dutch migration discourse from multiculturalism toward assimilation (Slootman and Duyvendak, 2016). For queer refugees, this pressure to assimilate presents a paradox (El-Tayeb, 2012). When queer Muslims assimilate into “Dutch national culture,” they risk reproducing an emancipation discourse of being “saved” by an “advanced society”. However, when they resist assimilating and reproducing this discourse, they are perceived as “backward” and thus incompatible with “Dutch national culture” (Slootman and Duyvendak, 2016).

This study examines the resisting and unsettling potential of art-practicing queer refugees in relation to hegemonic ways of othering they encounter. McGregor and Ragab (2016) argue for the transformative capacity of a variety of art practices on an individual level, “providing a creative space for exploration and expression of identities” (pp. 7–8). Art thus provides refugees the opportunity to express and transform their experiences of there and here into artistic expressions (McGregor and Ragab, 2016) as well as the opportunity to reconstruct one's identity (Beech, 2011). We claim that such reconstructive potential is particularly relevant to queer refugees because of their specific exilic (in-between) positioning. At a community level, a sense of communal learning and reflexivity can be encouraged via symbols, music, dance, and visual arts (Turner, 1979). At a societal level (queer, refugee), activists can use pictures, songs and other means to communicate outside of conventional language, to unsettle hegemonic discourses in playful non-discursive ways (Young, 2001).

Despite the reconstructive potential of art practices at different levels, the role of art in the social inclusion of refugees, especially queer refugees, remains understudied (McGregor and Ragab, 2016). This study therefore explores how art-practicing queer refugees challenge and unsettle various hegemonies at individual, communal and societal levels. Our aim is to fill this gap by, first, identifying the specific hegemonies in relation to and including their impacts on the lives of queer refugees, and second, exploring the ways in which queer refugees can unsettle these hegemonies based on empirical examples.

In this study, we concentrate on queer refugees' narratives of strength and resilience (instead of solely focusing on their vulnerability) that challenge hegemonic perceptions, while still acknowledging their struggles. We chose an intersectional and feminist approach that focuses on knowledge co-creation *with*, rather than *about*, queer refugees. Due to the COVID-19 pandemic, we used online/digital methods, combining biographical interviews with an arts-informed (Lenette, 2019) participatory component. Eleven queer refugees were interviewed via the web application Zoom and invited to participate in an artistic knowledge co-creation: *Art for Change* (AFC). Ten of those interviewed participated in AFC and created remunerated artistic works concerning their experiences during the pandemic that were then published on our website. Both the interviews and artistic works were considered as data. The leading question of this research was: *(How) can art-practicing queer refugees unsettle hegemonies in the Netherlands?* To answer this question, we first discuss power and agency and then zoom in on the specific impact of Dutch hegemonies on queer refugees. We then elaborate on differentiated forms of agency practiced by art-practicing queer refugees and the ways their agency challenges and unsettles Dutch hegemonies on micro, meso and macro levels.

## Theoretical Framework

### Hegemony and Normalizing Power

Coercive domination is a form of “hard power” that entails forcing others to act in a particular way (Wade, 2002, p. 203). Hard power tends to be overt and self-explanatory. For instance, laws criminalizing homosexuality can be considered an overt form of hard power. In contrast, control via “intellectual and moral leadership” was coined by Antonio Gramsci as hegemony (Gramsci, 1971, in Wade, 2002). Wade (2002) describes hegemony as soft power or “the ability to make others want the same thing as yourself, as distinct from hard power, the ability to force others to give you what you want” (p. 203). Hence, hegemony entails the ability to create a belief that the system of rule is fair and beneficial for both the dominant and the subordinate group (Wade, 2002). This belief makes it harder to envision alternatives or to think critically because it is taken for granted.

Michel Foucault conceptualizes power differently. Foucault argues that power is not about domination but rather about routinization and normalization (Foucault, 1978). One is not with or without power, but power is produced, transformed, weakened or strengthened in every relation by dominant and subordinate groups and individuals alike (Foucault, 1978). Thus, Foucault (1978) explanation of normalizing power differs from Gramsci (1971) conceptualization of hegemony, which understands hegemony in its relation between a dominant and subordinate group. However, normalizing power and hegemony share similarities in the sense that both are subtle and covert and cannot be pinpointed to a single source. Both concern internalizing processes about beliefs in the system and what is considered “fair and appropriate” (Wade, 2002, p. 201).

Despite this differentiation, most authors use a combination of hegemony and discourse to emphasize two interconnected aspects of power: a certain form of dominance and acceptance of the status quo and the aspect of normalization. Young (2001) uses the term hegemonic discourses as “a system of stories and expert knowledge diffused through the society, which convey the widely accepted generalizations about how society operates that are theorized in these terms, as well as the social norms and cultural values to which most of the people appeal when discussing their social and political problems and proposed solutions” (Young, 2001, p. 685).

An example of a hegemonic discourse is the othering of members of non-privileged groups (Ghorashi, 2018). These othering processes are situated within historically shaped context-specific discourses that normalize hierarchical categorizations of the self (as superior) and others (as inferior) (Ghorashi, 2018).

### Hegemonic Discourses Regarding Queer Refugees in the Netherlands

To begin, we introduce a dominant Dutch discourse regarding (forced) migrants without the additional intersection of a queer identity. This discourse concerns the hierarchical differentiation of (voluntary and forced) migrants in the Netherlands. Around 1960, the need for inexpensive labor in the Netherlands led to the temporary invitation of low educated migrant workers, particularly from traditional parts of Turkey and Morocco (Ghorashi, 2018). Refugees who arrived in the 1980s were seen as being in need of temporary humanitarian help and were thus also not expected to settle permanently (Ghorashi, 2018). Until the late 1980s, Dutch policies were therefore aimed at migrant communities maintaining the culture and language of their country of origin in anticipation of their expected return, and policies were aimed at facilitating that return (Slootman and Duyvendak, 2016). Toward the end of the century, however, it became evident that most migrants were settling permanently in the Netherlands (Slootman and Duyvendak, 2016). Even though policies had never intended integration, sentiments emerged that “the ‘integration’ of immigrants failed” (Slootman and Duyvendak, 2016, p. 56) because migrants had never adopted the Dutch culture and language. Therefore, since the 2000s, policies have increasingly shifted toward pressuring migrants to assimilate (Slootman and Duyvendak, 2016). This then added another layer to the already existing hierarchical differentiation between the Dutch self and the migrant other, namely, the assumption that migrants were unwilling to integrate. Consequently, the language in such discourses harshened and policies forced migrants and refugees to assimilate. At the same time, a sense of non-recognition and non-belonging in different generations of migrants and refugees grew (Ghorashi, 2018).

In the case of queer refugees, additional components exist within the hegemonic Dutch discourses. Bracke (2012) identifies homonationalist, homonostalgic and homonormative othering discourses. The first component, homonationalism, concerns the idea that gay rights and sexual freedom are incompatible with Islam. This narrative became prominent after the murders of two outspoken public figures, Pim Fortuyn and Theo van Gogh, who both argued that sexual freedom in the Netherlands was “under attack” (Mepschen et al., 2010).

The second component of the othering discourse is “homonostalgia” (Wekker, 2009), which is the dominant idea that western acceptance of homosexuality corresponds with having achieved an advanced stage of modernity. Muslims, among others, are then depicted in opposition to such “modernity” as homophobic and “behind” in terms of emancipation (Wekker, 2009). This dominant way of othering frames “a modern self” vs. “a traditional other,” resulting in a kind of nostalgic longing for an imagined time in which gay liberation was not “threatened” by the arrival of Muslims. Furthermore, “nostalgia” frames racist inequality narratives and feelings of superiority as innocent, not fitting the Dutch progressive self-image (Wekker, 2009, in Bracke, 2012).

The third component is the “new homonormativity,” a term coined by Duggan (2012). Duggan argues that homonormativity can be defined in terms of neoliberal politics that aim for assimilation into heteronormative values and practices in order to depoliticize gay culture through consumerism and a focus on domestic normative life (Duggan, 2012). For instance, heteronormative values such as marriage and monogamy are privileged and replicated by cisgender homosexuals (Halperin, 2012, p. 441). As a result, “gay culture” is depoliticized and “Dutch gay identity does not threaten heteronormativity, but in fact helps shape and reinforce the contours of ‘tolerant’ and ‘liberal’ Dutch national culture” (Mepschen et al., 2010, p. 971). Hence, homonormativity concerns a western dominant view on how homosexuals are considered “properly gay” by not being “too political” or critical of heterosexual norms (Mepschen et al., 2010).

Consequently, there are at least two main meanings of the term “queer”. One is a depoliticized definition in which queer is used as an umbrella term for non-heterosexual identities (Jagose, 1996). The other is a “political” definition of queer as an (ideological) non-conformity to stereotypes concerning gender and sexuality, thus resisting both heteronormativity and homonormativity (Vijlbrief et al., 2020). Accordingly, there is a tension within the LGBTQ+ community in which sexuality and gender norms exist in a “sex hierarchy” (Rubin, 1984/1999). In Gayle Rubin's concept, individuals who do not assimilate into homonormativity, such as “transsexuals, transvestites, fetishists, sadomasochists, [and] sex workers” (Rubin, 1984/1999, p. 151), are considered to be at the bottom of the sex hierarchy.

Discourses on migrants/refugees, as well as homonationalist, homonostalgic, and homonormative othering discourses, therefore have a common concern of creating a self-other hierarchy. Within this hierarchy, an additional differentiation is made (an othering within an othering) in which queer refugees are perceived as (almost) incompatible (so at the bottom of otherness), while they are simultaneously pressured to assimilate. This creates a paradox for queer refugees: when they assimilate in a “proper Dutch way” by internalizing “the Dutch national culture” (Slootman and Duyvendak, 2016), they risk reinforcing the dominant narrative that western modern values liberated the “victim backward other”. However, when they do not assimilate and they express identities that are not considered “authentic” according to dominant standards (e.g., Islamic queers or “politically” queer), they are accused of being neither “properly Dutch” nor “properly Muslim” nor “properly gay” (El-Tayeb, 2012). Hence, the question we engage with in this article is whether and how art-practicing queer refugees can unsettle such hegemonies in the Netherlands.

### Liminality

To answer this question, we start by exploring the importance of the concept of liminality to understand the in-between position of queer refugees in our study. Liminality is described as an ambiguous transition state, usually to a higher personal or societal level, in which individuals or groups “are neither here nor there; they are betwixt and between the positions assigned and arrayed by law, custom, convention, and ceremonial” (Turner, 1969, p. 95). Liminality is characterized by uncertainty due to the temporal suspension of norms and behaviors (Turner, 1969; Van Gennep, 2019).

In the case of queer refugees, we conceive liminality in two main aspects: their migration background and their SOGI. First, migration procedures are liminal due to their transitory character in which one is between one's former and new country (Manjikian, 2014; Ghorashi et al., 2018). Second, in terms of SOGI, liminality can be used to explore the challenges due to the specificity of their intersected identity positionings. Beech (2011) explains identity construction as a process in which “[t]he co-construction is enacted in the interplay between an individual's “self-identity” (their own notion of who they are) and their “social-identity” (the notion of that person in external discourses, institutions and culture)” (Beech, 2011, p. 1). He argues that “liminality can be defined as a reconstruction of identity, in which the sense of self is significantly disrupted, in such a way that the new identity is meaningful for the individual and their community” (Beech, 2011, p. 3). Monro (2005), then, considers transsexuality as a liminal space outside the gender binary. We propose that liminality is not limited to transsexual people alone but applies to queer and gender-nonconforming people more generally. In this article, we thus acknowledge queerness and refugeeness as liminal spaces in which there is potentially room to (re-)construct queer refugees' multiple identity markers in a personalized way, as opposed to forcing them to assimilate into societal norms.

### Agency in Liminality

Although Beech (2011) stresses the psychological burden that may accompany states of liminality, such states may also be “full of experiment and play” (Turner, 1979, p. 466). Victor Turner argues that liminality encourages reflection, which can be considered a form of agency (Turner, 1979). Agency refers to the human capacity to act: a capacity shaped by (non)available possibilities and resources within the social world and its discourses and practices (Björkdahl and Selimovic, 2015).

Turner (1979) considers reflection as a source of agency that corresponds to what Judith Butler calls “discursive agency,” the ability to name one's subjection to soft hegemonic power (Butler, 1997, p. 127). Discursive agency means practicing a deconstruction of social hegemonies via acts of public misappropriation in order to counter conventional meanings (Youdell, 2006). Thus, discursive agency concerns the ability to deconstruct fixed categories of oneself and another and to unravel contextual specificities (Ghorashi, 2017).

There is also a creative form of agency that entails “a play of ideas, a play of words, a play of symbols, a play of metaphors” (Turner, 1979, p. 466). This non-discursive and creative liminality corresponds to Björkdahl and Selimovic's (2015) feminist approach to agency. These authors deconstruct traditional approaches to agency, and in addition to promoting discursive agency, they stress the creative aspect of agency that is often neglected in other approaches (Björkdahl and Selimovic's, 2015). Creative agency has transformative qualities because it challenges and resists existing oppressive structures in a creative and (sometimes) even unintentional way (Björkdahl and Selimovic's, 2015).

### Art Practices' Potential in Unsettling Hegemonies

Having conceptualized agency as both discursive and creative, we now turn to art practices as potential ways of expressing agency in relation to hegemonies. In addition to art as an aesthetic practice, it can also have more political implications that manifest themselves on all societal levels (Malik et al., 2020). Studies have shown that art practices can have therapeutic properties: promoting self-esteem, facilitating emotional self-expression, aiding the processing of traumatic events and helping express multiple identity markers concomitantly (McGregor and Ragab, 2016). We argue that such therapeutic potential can be seen in light of Beech (2011) liminality as identity construction. In fact, queerness, migration, and art practices can all be considered liminal (Turner, 1979). Thus, on the individual level, practicing art may allow the reconstructing of multiple identity markers and facilitate transforming queer refugees' paradoxical experiences into an integrated, personalized, unconventional identity.

In addition, Turner (1979) argues that performative arts affect audiences as forms of “plural reflexivity” (p. 465), which he describes as ways in which communities think about, see and present themselves through signs, symbols, music, dance and visual arts, thereby strengthening a sense of community. In the context of queer refugees, plural reflexivity can be understood as sharing and negotiating one's paradoxical experiences and queer identities through art within, and outside, the (queer, refugee, artist) community.

On a societal level, Turner argued that *public* liminality has often been regarded as threatening by powers representing the dominant established group because “[t]he powers of the weak—to curse and criticize—set limits on the power of the strong—to coerce and ordain” (Turner, 1979, p. 465). He claims that public liminality cannot be considered solely as an emotional outlet but rather as subversive proposals. We suggest that such subversive demonstrations can be considered as a form of activism.

Due to the subtle and masked nature of hegemonic power, the activist is often skeptical of deliberative approaches with those in power (Young, 2001). Deliberation forces one to speak within the language of the present hegemonic discourse. The problem is that the hegemonic discourse masks unjust power relations and is on the side of the dominant group (Young, 2001). So, rather than arguing in a deliberative manner, “the activist's goal is to make us wonder about what we are doing, to rupture a stream of thought” (Young, 2001, p. 687). Activists, according to Young (2001), do this by using “non-discursive means” such as “pictures, song, poetic imagery, and expressions of mockery and longing performed in rowdy and even playful ways aimed not at commanding assent but disturbing complacency” (p. 687).

In this section, we have elaborated on theoretical notions related to power and agency. We first presented the layered aspect of the hegemonic Dutch discourse concerning queer refugees. Then, by using the frame of liminality, we argued for a broader approach to agency, including not only discursive but also creative forms of agency. We also discussed various ways that hegemonies can be unsettled on individual, communal and societal levels. In the empirical section that follows, we show the multi-level and layered forms of agency performed by the art-practicing queer refugees who participated in this study.

## Methodology

To study how queer refugee artists can unsettle exclusionary hegemonies, we chose a feminist intersectional approach. An intersectional approach focuses on understanding and describing power dynamics in social inequality via multiple intersecting levels in the human experience that influence one another, such as gender, sexuality and ethnicity (Gray and Cooke, 2018). For unsettling dominant hegemonies, we suggest that the most suitable research approach is a qualitative emic inductive approach: that is, a context-specific bottom-up understanding from the participants' perspectives (Tracy, 2013). We collaborated solely with queer refugees in order to give more space for their voices, as opposed to including voices of “experts in the field” who do not identify as queer refugees. In addition, we aimed to create a space to acknowledge and establish a (societal and academic) platform for the various ways in which participants express agency (discursively and creatively). We argue that such an approach is salient, not only for increasing our understanding of participants' perspectives, but also for adopting societally engaged and relevant research practices.

### Feminist Approach

Feminist research critiques the epistemological and ontological claims of objectivity and rationality, while arguing that all knowledge is situated and socially constructed and therefore subjective and partial (Undurraga, 2012). Feminists have traditionally intended to empower women by centering them in their analysis (Kingston, 2020) as well as to give voice to members of other marginalized groups (Tracy, 2013). Furthermore, feminist approaches expect that the produced knowledge should have (practical) use and they aim for societal change as opposed to knowing in its own right (Tracy, 2013).

A feminist approach suits our research design best for three reasons. Firstly, we share the epistemological and ontological position of reality and knowledge being socially constructed (Undurraga, 2012). Secondly, in the process of knowledge production, we aim to shift power toward a more horizontal way of collaboration *with* participants as opposed to a more hierarchical research design in which the research is *about* participants. A feminist approach, with its attention to power relations, has the potential to unsettle more conventional research methods in which discourses about the “marginalized” are being reproduced (Lenette, 2019). Thirdly, considering our aim of acknowledging and establishing a (societal and academic) platform for the various ways in which participants express agency, we follow a tradition of knowledge production that strives for practical use and societal change (Tracy, 2013).

In addition, feminist scholars argue that researchers should be reflexive about their position and power to ensure the validity and qualitative rigor of their research (Guillemin and Gillam, 2004; Gioia et al., 2013). Reflexivity entails awareness and transparency regarding the way in which one's position and interactions as a researcher influence the process and the produced knowledge (Caretta and Riaño, 2016).

The first author's positionality in relation to our research is shaped primarily by their queer identity, second-generation migration background, experiences growing up in different foster families and professional background in acting and theater; they had an appointment as a student assistant at the time of the research. The second author is a cisgender, heterosexual woman with a migration background, a partially completed professional education in classical ballet and two masters; she is currently a PhD candidate. The third author, who is a full professor, is a cisgender, heterosexual woman with a refugee background. During the research process, we, as authors, not only engaged with the data but also reflected on our specific positionalities in relation to each other and to the data and its interpretation. In particular, the first authors' partial insider position enabled them to gain access to, and built trust with, participants much more easily. In fact, many participants talked to them very openly about sensitive issues.

### Research Methods and Sampling During COVID-19

In January 2020, prior to COVID-19 physical distancing measures, we intended to use participant observation, biographical interviews and discourse analyses as research methods. However, once physical distancing measures were put in place in March 2020, physically meeting with participants became impossible. We then shifted toward online/digital research methods, namely, biographical interviewing (via Zoom) and an arts-informed (Lenette, 2019) participatory research component in which participants were asked to create a remunerated artistic work about their experiences with COVID-19 for an online platform: *Art for Change* (AFC). The objective behind this shift was to continue the research while also using it to help mitigate the socioeconomic and emotional impacts of the pandemic by supporting queer refugees (emotionally and financially) in overcoming some of the challenges they faced. These challenges entailed losses of income due to the cancellation of events, grief due to the sudden lack of physical connection with members of their community, and distress regarding the refugee crises around the globe. AFC also aimed to challenge dominant images of refugees as victims, unimaginative and dependent, by creating a platform for refugees' stories and art about their challenges and resilience during COVID-19.

Asking potential participants to share their experiences of COVID-19 in conversations, and to take part in a remunerated artistic knowledge co-creation via AFC, seemed to make them more interested and inspired to engage in this collaboration. Nonetheless, we still had to gain trust and build relationships. Offline meetings were replaced by online meetings via chats in social media, followed by audio or video calls, during which potential participants decided whether they would agree to collaborate. Before COVID-19 measures were put in place, we had interviewed four participants. Three of these interviews were conducted offline in the participant's house, and one was conducted online. After COVID-19 measures, we conducted eight additional interviews online via a licensed secured connection in the web application Zoom. Most interviews took between 90 and 120 min.

We used purposeful sampling (Tracy, 2013) and aimed for participants who fit within the following parameters: identifying as queer, having a forced migration background, practicing art in the broadest sense and living in the Netherlands. Each different parameter significantly narrowed down the number of potential participants. However, due to the first author's experience in art and co-organizing safer space events for queer and trans people of color, we felt confident to find enough participants. Fortunately, the first author had already visited several events in which art-practicing queer refugees were participating before COVID-19 measures were put in place. Through those events, we were able to recruit eight research participants (who either participated in the events themselves or were connected to us by other participants/organizers). The first author personally knew three additional participants from the queer community. Eventually, ten of the eleven interviewees participated in AFC.

Participants had diverse cultural backgrounds and origins (i.e., Morocco, Iran, Afghanistan, Brazil, Belarus, Congo and Burundi) and their ages ranged from 28 to 42 years old. They were also diverse in gender identity and expression: five participants identified as male, four as gender non-binary (using they/them pronouns), one as female, and one as non-binary femme. Although we consider this a diverse gender variety, only two participants were assigned female at birth. Of those two, one was male and one was non-binary. Thus, seven participants were transgender (two binary, five non-binary). The overrepresentation of some identity markers was due to the fact that these were more visible to the first author. Given that one cannot or should not assume someone's SOGI or migration background (or even art practices), asking about such personal issues is sensitive and can be problematic, particularly, before having established trust.

#### Biographical Interviews

We chose biographical interviewing because of its storytelling capacity, which is similar to art practices in the sense that both concern telling stories about (one's) life. Biographical interviewing created space for participants to tell their stories in ways they saw fit. All interviews were conducted by the first author, who is themselves a queer artist (using they/them pronouns) with a second-generation migration background. To establish trust, rapport, reciprocity and conversation flow, the first author would begin by explaining the context of the interview and sharing parts of their own biography (Undurraga, 2012), knowing that “[i]nsider/outsiders also have to be open and expose themselves, their histories, and stories if they are asking others to critically reflect upon sometimes painful individual and familial histories” (Schensul et al., 2008, p. 132). They then asked an open question about how the participant became the person they are right now and what led them to their art practices. If the conversation did not flow organically or cover all the Research Topics, the first author would ask prepared questions. The interview topics concerned the following: country of origin (growing up); migration story; early perceptions of the Netherlands and whether those had changed over time; feelings and experiences regarding the Dutch LGBTQ+ community; development of their art practice(s); challenges, strategies and dreams concerning their art practice(s); messages in art; activism; and queerness.

#### Art for Change, an Artistic Knowledge Co-creation

In addition to the interviews, the artistic works created by AFC participants were considered data for this research. The artistic works consisted of a song, written texts, a “decolonizing” deejay set, Grindr poetry, video poetry, performance, drag and graphic design (see Table 1). Adopting an arts-informed participatory approach was important for several reasons. Involving participants as creative knowledge co-producers potentially enables critical reflection on and unsettlement of power (im)balances in a more democratic research process (Lenette, 2019). Such an approach is emancipatory in the sense that self-determination is central, objectification of forced migrants is reduced, and situated knowledge based on lived experience is valued (Mahn et al., 2019). By asking the artists to create remunerated artistic works, we aimed to shift more power toward the participants and convey that we took participants (and their perspectives, knowledge and works) seriously as partners and artists, thereby validating their knowledge, creativity and experience.

**Table 1:** Artistic works published in Art for Change^1^.

**Artistic work**	**Title**
Song	The Voice Of The Queer
DJ Set and text	Mamakil's (decolonizing) DJ set
Performance	Labyeeka Ya Hussein
Visual poetry	Corindr
Audio play	De Wortels (The Roots)
Vodcast (2 episodes)	TrannyCast
Video poetry	Venusus/Lockdown My Spine
Video/drag	Grizolda Storm
Text	Jouw Taal Is De Mijne Niet (Your Language Is Not Mine)
Graphic design (6 designs)	Listen To My Eyes

In addition, the output of arts-based projects can be communicated to and inform wider communities as well as the academic world (O'Neill et al., 2002). More importantly, art practices offer a means of imagining beyond the limits of language (or playing creatively with language) via more sensorial, visual or emotional levels (O'Neill et al., 2002). Participants may feel more at ease communicating beyond the level of language, especially in cases where speaking in a second, third or fourth language is a challenge in itself (Karimi, 2020).

Finally, in line with Oliveira (2019), we argue that collaboratively producing knowledge allows for a fuller understanding of complex issues in a process where critical awareness can be developed. Co-creative research methods largely deal with reflexive processes, affecting both the participants and the researcher (Oliveira, 2019). These reflexive processes are similar to those in art practices, particularly art practices concerning social justice that are process based rather than focused on an end result. The process of critically developing an idea, choosing material and deciding whether and how to share it with the world is in itself salient (Dewhurst, 2010).

As researchers, we engaged reflexively with issues related to the research and the presentation of the data. After having thoughtful discussions, we decided to reimburse research participants for their art works because of their income losses due to COVID-19. This decision may have influenced their choice to participate, considering that some were initially unresponsive regarding whether to collaborate. An unintended consequence of our decision was an enlarging of the power distance between the participants and us as researchers. Even though we tried to shift power to the research participants, several asked for advice and approval of their artistic works, as if we were “in charge”. We tried to balance these requests by stressing that participants had full freedom and co-ownership, while also trying to help take away their uncertainties by giving some suggestions.

A second consideration was the issue of anonymity. Most participants wanted to use this opportunity to showcase their works with their own (artist) name on a platform (the research website) to reach outside their communities. Due to the university's ethical requirements, recognizability can be problematic and is thus prohibited. With the aim of acknowledging participants as artists, while also protecting their anonymity, privacy, confidentiality and vulnerability, we chose to split the project into two parts: (1) the artistic works in which participants could choose to use their own (artist) name, a pseudonym, or no name at all (the risks and possibilities of each choice were thoroughly discussed with each participant individually) and (2) the biographical interviews in which all participants were pseudonymized. For reasons of anonymity, we decided not to link the specific works and cultural backgrounds to the artists. In addition, in this article, we sometimes refrain from using participants' pseudonyms after quotations in cases in which their quotes could be traced back to their artistic contributions.

### Data Analysis

All interviews were recorded and transcribed verbatim, while pseudonymizing all names of people (work), places and projects. Exceptions are the countries of origin and the residing Dutch cities. All data was stored in a secured online repository protected by a password and accessible solely to the research team. The interviews, as well as the artistic works, were analyzed iteratively, alternating “between emic, or emergent, readings of the data and an etic use of existing models, explanations, and theories” (Tracy, 2013, p. 184). We first created a code tree related to the research questions, theoretical framework, and interview questionnaire. We then tried to be extra attentive and open when approaching the data. The artistic works were coded similar to the interviews, with eight out of ten works partially consisting of written or spoken texts. After the second round of coding we visually organized codes, checked co-occurrences and added connections between codes and written memos. In organizing and visually mapping the data, we noticed many contradictions. At first, we considered that participants may differ too much in individually faced hegemonic challenges to analyze them as a group. Then, we organized the contradictions thematically and discovered that they could actually be distinguished into four main paradoxes, namely, the paradoxes of “representation,” “desirability,” “belonging” and “participation”. Subsequently, we discussed and identified several forms of agency, and distinguished three overarching themes for how participants express agency: queerness, collectivity and art practices.

## Empirical Findings

This article focuses on Dutch hegemonic challenges as narrated by the participants in our project and the ways they engaged with those challenges. The data shows that hegemonic challenges pose a variety of paradoxes for queer refugees. We will show how participants resist these paradoxes and how they position their art practices and queerness in relation to them. Finally, we will discuss the findings in light of participants' agency.

### Hegemonic Challenges

Hegemonic challenges are more insidious than overt challenges because they are forms of “soft power” (Wade, 2002). We identified three themes regarding how hegemonic challenges manifest themselves on three different levels: hegemonic discourses (macro), institutional dominance (meso), and interpersonal experiences of violence (micro).

At the macro discursive level, some participants described how Dutch natives were sometimes shocked when their expectation of “poor queer refugees in need” was disrupted by, for instance, beautiful clothes participants brought from their countries of origin. Moreover, some of their Dutch native contacts expressed feelings of unfairness.

I lost my, like, two, three relationships just because they came to my house and they said, you are just the refugee, you get *uitkering* [social benefits] and your house is much nicer than my house. My house is empty and you live in a social house. (Arash).

Narratives related to the meso level address issues at an institutional level. This theme relates to art, media, educational and governmental institutions that have controlling power in terms of including or excluding people. Such institutions control what and who is represented, according to what they believe to be “right” or “fair and appropriate” treatment, artistic works or behavior. Examples range from bureaucratic procedures regarding access to arts funding to exclusive structures in cultural events, such as gay pride, and media representation.

Finally, micro level narratives of exclusion relate to individual interpersonal experiences of violence based on homophobia, transphobia or racism. For instance, participants mention being judged or labeled and encountering (verbal, physical, online) harassment or assault due to their SOGI or migration background.

**Figure 1:**
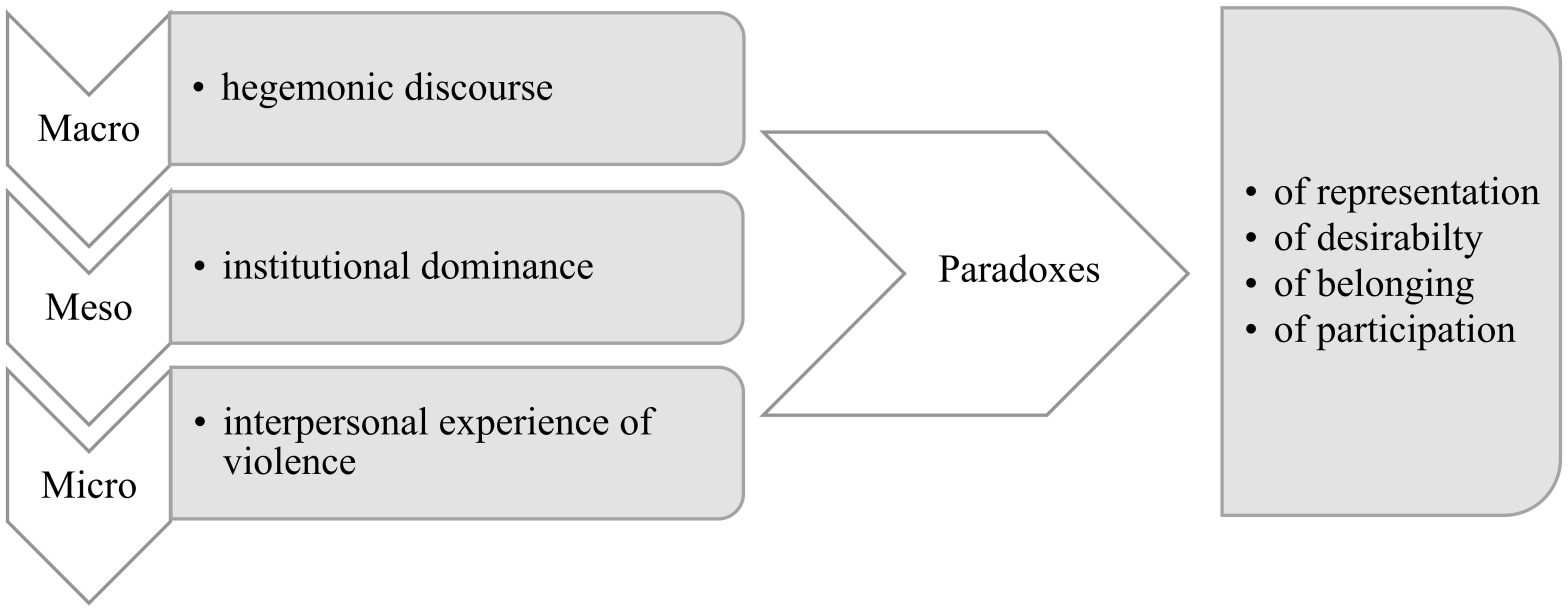
Three themes regarding how hegemonic challenges manifest themselves on all societal levels, including the paradoxes they create for queer refugees.

In analyzing various hegemonic challenges, we discovered four paradoxes: the paradoxes of (1) representation, (2) desirability, (3) belonging, and (4) participation (see Figure 1). Although separating these paradoxes implies a certain strict or definitive order, in reality, the paradoxes are intertwined and multilayered. However, presenting these paradoxes separately helps us expose the complex situations participants find themselves in due to their different intersecting identity markers.

#### Paradox of Representation

Participants mentioned a lack of representation of marginalized identities in the media and in society at large. For them, representation is predominantly one-sided and stereotypical, as opposed to multifaceted and well-rounded. In other words, participants argue that marginalized identities are often depicted as either “very good” or “very bad”.

When I used to watch the late-night news, I saw people that I could identify with as either celebrities or bad people. So racism is basically handed on a platter. (Calvin) [Translated from Dutch]

Even though participants reported an increase in certain kinds of representation of marginalized identities in society, according to them, this does not necessarily result in increased societal inclusion or equality.

So I see a huge change in art and in classifications where a certain image of society needs to be portrayed. We all know that there are also many commercial purposes and that there is now also a kind of collective consciousness. *Now*, if you don't do it, you will be pointed out. But a lot of people do not understand the essence of what you do. And the fact that you can watch a Prada show of 46 models, and 18 are Black, does not mean that Black women in […] the more normal, ordinary layer of society are more accepted. (Calvin) [Translated from Dutch].

Calvin, who is Black, is a professional dancer and model. When asked whether he could imagine a career outside of art, he responded negatively. His ambition has always been to become “the Black greatness”. His statements suggest that despite increased representation, characterizing marginalized people as being either very good or very bad is still very present in societal discourses. This seems to leave little room for individuals to construct their own (and more nuanced) identities.

Some participants argued that achieving excellence is not only based on one's self-image but also forced from outside, as people of color are judged more harshly in comparison to White people. This is in line with the concept of “tokenism” as used by Ghorashi and Sabelis (2013). Tokens are individuals who are perceived as representatives of minority groups, specifically in places (e.g., work) where there are few other members of the same minority group. Tokens are often subsequently burdened by the pressure of representing a “good example”. In similar fashion, participants in this research described a fear of the burden of having to represent a larger community.

I'm only representing myself. I'm not representing anyone. I'm not. […] OK, don't put it on me. And I don't want to [be a] leader. Like, you know, yeah, so this was also a struggle I was facing in my life. (Shif)

In summary, although some participants saw an increase in the representation of marginalized identities in the media and in societally respected positions, they argued that such representation still remains stereotypical and lacks nuance. Additionally, some participants felt that minorities are being judged harsher than majorities. Differentiations of marginalized identities as either very good or very bad thus seemed to leave little room for nuanced and more realistic representations. This dichotomy not only limits individuals from developing more nuanced identities but also keeps societies from becoming more inclusive of difference.

#### Paradox of Desirability

Similar to the paradox of representation is the paradox of desirability. Due to specific “looks” or “behaviors” related to ethnicity and gender expression, queer refugees experience being simultaneously desired and undesired. Some participants are fetishized (as Muslim or Black, for instance) by some, while feared or simply rejected by others (for being Muslim or Black). Some participants experience being feared because they “look Muslim” (Shif). Others are rejected because they wear makeup or are perceived as being too feminine. Participants are affected by these forms of othering in their daily interactions, particularly on gay dating apps.

The biggest problems are in the community. […] We have racists there. You have, just if you check the apps, that's a good example. Like, if you are Asian, if you're Black, if you're this, don't text me. If you're feminine, if you wear makeup, don't text me. So why do you have to mention that actually? And then it does send you a message, “Hey, oh, you look beautiful. But are you feminine or manly?” You know, I hate this kind of conversation. (Arash)

Furthermore, some participants are regularly asked if they are sex workers on gay dating apps. This can be considered a form of objectification and commodification (Constable, 2009), which further reproduces the narrative of “the ethnic other” as being different or inferior.

Yet, rather than being discriminated against or excluded, other participants feel that they are desired or to some extent even fetishized by the host LGBT community.

No, I'm Persian, we don't experience that [exclusion or discrimination within the LGBT community]. [laughs] Don't take it as a point of ego, but this is just what I've experienced. People are, people tend to, Europeans tend to or westerners tend to be more interested after they hear that I'm Iranian. (Maheen)

Thus, the paradox of desirability concerns queer refugees being considered as the other and being simultaneously feared (or rejected) and fetishized (objectified and desired). Hence, the perception of the other as being fundamentally different and inferior is further reproduced. This is not so different from the paradox of representation and its stereotypical and polarized representations of very good and very bad.

#### Paradox of Belonging

The paradox of belonging is complex due to the many overlapping intersecting elements of participants' identities. This paradox concerns participants' feelings of non-belonging, or lack of acceptance, in any of their respective communities. For instance, participants are considered not Black enough for one community but too Black for the other.

So at certain moments, it's confusing, like in high school. There I experienced that even Congolese people [in Belgium] said you don't belong with us. You're not one of us. Because, first, they had doubts about my sexuality, and I wasn't raised with the culture. So you're not Black enough. But then at the same time, you're too Black. (Calvin was adopted at birth by a White Belgian family) [Translated from Dutch].

Calvin thus experienced exclusion due to the intersecting combination of his sexuality, his “White” upbringing and his Blackness.

Another example involves being queer and being Muslim. The following text is an excerpt of a voiceover from a performance by one of the participants in AFC. The text exemplifies the paradoxical feelings of being queer, feminist, and Muslim at the same time. This corresponds with El-Tayeb (2012) concept of queer Muslims in Europe, which argues that queer Muslims are perceived as neither “proper queers” nor “true Muslims”.

People have always insisted on asking me, no shame, are you really Muslim and at the same time you're a man? Are you really Muslim while wearing those clothes? Don't you think you are contradicting yourself? Are you really Muslim and practicing feminism? Are you really Muslim and queer? How can you be Muslim wearing those faggoty feminine clothes? […] You contradict yourself being queer and Muslim. Same as Islam and feminism. Did you really read the Quran well? (Participant)

In addition, despite the hegemonic claim that Islam is incompatible with the perceived advanced, emancipated (Dutch) society, many participants considered themselves *more* emancipated than members of the Dutch LGBT community. Ghano, for instance, feels that they (personal pronoun) do not belong to “White gays” because the more normative, White gay and lesbian individuals are “not as political” as Ghano perceives themselves. In the end, participants spoke of their experiences (or feelings of) triple marginalization.

Because it adds the racism into it, with the intersectionality of the struggles, becoming a refugee, becoming like a minority within the minority within the minority. (Ghano)

Triple marginalization in this example refers to *a minority* (a refugee of color in the host society) *within the minority* (a refugee of color in the host LGBT community) *within the minority* (a queer refugee of color as opposed to a homonormative member of the host LGBT community).

The intersection of (some) queer refugees' identifications thus reinforces exclusion in multiple aspects. This results in participants experiencing not belonging to any community due to a combination of characteristics that are perceived as conflicting by different communities. Central to this paradox is a perceived “liberated, emancipated Dutch culture” in conflict with the perceived “unemancipated other,” even though (some) queer refugees perceive themselves as more emancipated than host (cisgender) gay community members.

#### Paradox of Participation

Our interviews have shown that on the one hand, participants have an intrinsic desire, and experience societal pressure, to participate in society. Participants were often eager to work or study, and they expressed the wish to integrate and follow their ambitions. They also anticipated feelings of freedom after the overt coercive oppression experienced in their countries of origin. On the other hand, participants' societal participation is highly dependent on, and in fact often inhibited by, specific expectations, requirements or paradigms of societal (art, educational, medical, governmental, and funding) institutions. They are thus often not free to participate in the way they would like to.

So, and I am still linked to the municipality. They choose for me. And even things I dream of, it cannot be your dreams. It's going to be the bureaucratic dreams. […] I don't think the institutions is for artists like me. I was just trying to find my way to get integrated into society, but the society don't want me to get integrated. (Amina)

Such (unspoken) expectations of societal participation are exemplified by the situation in which a participant was rejected by an art school for not being “neutral enough”. The participant in question is non-binary trans, queer, activist and Muslim. There thus seems to be an expectation that art school applicants should be “neutral,” and apparently, people assumed that the applicant in question could not be neutral. Additionally, there seem to be assumptions about what is appropriate to be presented in art or what kinds of topics minorities should focus on. Participants spoke of funding bodies not supporting certain projects concerning taboo topics such as non-normative sex practices. Moreover, participants argued that they only get remunerated when they write within a narrow range of (queer, migrant) topics.

And I also think that the precariousness of a lot of young writers, especially of color, the precariousness in which they live, which also forces us to just write or respond, just to get that 250 or 300, just to get your rent paid. […] I then see other more established white male writers who have just been working on a book or a project for 5 years. […] I have a lot more to offer than my pain, you know? […] Tell me about your pain, you know? Your gender pain, your racist pain, your domestic violence pain. Pain pain pain, you know? And then you will be paid. (Participant) [Translated from Dutch]

It thus seems that refugees are often expected to represent themselves as the vulnerable, victimized, stereotypical other, while avoiding topics that are considered taboo or too political. They thus have to stay more neutral.

To conclude, the paradox of participation concerns queer refugees being expected (or even pressured) to participate in society, while the manner of participation is determined by host society institutions' (sometimes unspoken or contradicting) expectations, requirements or paradigms.

### Queer Refugees' Resistance

We now turn to how participants deal with, and position themselves in relation to, the hegemonic challenges described above. Although there are various ways in which participants do this, we focus on the three most salient themes for this article: namely, resistance via queerness, collectivity and art practices.

#### Queerness

The term *queer* was often used by participants both as an umbrella term to capture identifications outside of heterosexuality and cisgender identities (Jagose, 1996) and politically to resist hetero- and homonormativity in general (Vijlbrief et al., 2020). Some participants reasoned that queer as an umbrella term simplifies the complexities and fluid nature of identifications. The political meaning of queer, however, has far more relevance to most participants.

Oh, queer means a lot of things for me. […] Like being revolutionary. Revolutionary on the society, not accepting shit that society wants to put on you, […] [J]ust not responding to any norms at all regarding sexuality, regarding love, regarding family things, regarding anything, like, regarding religion. I think anyone, for me, like, anyone who does not accept this, how do you call it? Like pre-made thoughts, like question them, and creating their own revolution as a queer person as long as they are not discriminating others. (Shif)

Identifying as queer thus implies (actively) resisting various cultural norms. Queerness then becomes equivalent to activism; participants considered themselves activists due to their queerness. We argue that participants' collectivity and art practices are both embedded in queerness, as an important expression of agency and as a form of resistance.

#### Collectivity

Another important aspect of participants identifying as queer is that this identification implies feeling or being part of the queer community. In general, distinguishing oneself from more normative lesbian, gay, bisexual identifications seemed to be important for most transgender, queer participants. There seems to be a divide between homonormative LGB and non-normative TQ+ identities. In addition to distinguishing oneself from LGB identities generally, building or strengthening one's own specific (queer, migrant, refugee) communities was considered very important. Some explained it as having a more “relational” sense of identity as opposed to “a more individualistic identification”.

Giving and receiving community support was considered crucial after having to start over in a new country. Without such support, dealing with the many challenges in the Netherlands would have been almost impossible.

No, it was not mentally possible to cope, to do things alone. […] I like to meet people, to meet people, to talk to people and from there creating a personal connection and make the work happen. (Travequinha Safada)

Financial support provided by members of the community was also mentioned by participants.

Yes. It's like first, first community here who helped me. And trust me. And accept me. Yes, it's true. […] Yes. JH [mother of a drag family] helped me being creative. (Yakiv)

On an individual level, the collective thus provides key emotional, practical and financial support.

Additionally, according to several participants, also provides safer non-mixed spaces in which artistic and self-expression is encouraged. Examples of non-mixed spaces may be queer-refugees-only spaces or trans-only spaces. In such spaces, participants may no longer feel the burden of, for example, potential misgendering, harassment and other forms of violence. They may be more free from the pressure to explain themselves in terms of their gender or ethnic identity and more able to share and negotiate (similar) experiences and develop critiques, techniques and approaches.

So I think that is why for me, non-mixed spaces or performing those specific things with a specific group that intersects with my struggles is important and makes it just safe for me to do. (Shif)

However, there are also critical reflections on the notion of safer non-mixed spaces.

I recently organized an event around the idea of Black liberation. I invited all Black people […] there was one person I also invited who was not Black. […] [A] two-hour conversation was planned, of which at least half an hour was spent by people who complained, […] [that] we are not safe to speak […] [W]hat you need is a safe space. But […] I'm the only trans person in this event. So am I safe to talk to you guys about Black liberation? Are you safe for me? […] I'm also one of the most highly educated of them all. […] Is that a safe space for them? I come from a family that is very rich, super rich compared to what these kids grew up with. […] Most of the people in the group have all kinds of gender studies or sociology […] Is that safe? You know, what are we talking about? (Osa) [Translated from Dutch].

Osa prefers the term negotiated spaces to that of safe spaces and eloquently and critically reflects, via an intersectional lens, on the question: What is safe for whom?

Lastly, queer communities also organize activism, such as demonstrations. Such demonstrations involve collective and independent (from institutions) organization in which initiative and decision-making lie only within the queer migrant community. Further, some participants express the benefits of becoming more engaged and taking initiative as well as the importance of having resources circulating within the community to make collective actions possible. For example, the community organizes other benefits and events in order to raise funds for demonstrations, and it may use those funds to pay for train tickets for people from other cities.

To summarize, collectivity entails emotional, practical and financial community support, organized negotiated spaces (mixed or non-mixed) where people can feel free and relatively safe to express themselves (mundanely or artistically), and collective action (activism).

#### Art Practices

In this research, we identified five ways in which participants express agency through their art practices. First, participants said their art does not necessarily have to be connected to experiences of exclusion; it may also explore notions of beauty, humor or mundane topics. For instance, one participant explained their art was mostly about love, spirituality, spells for witchcraft and their cat. Although these seemingly banal expressions of art may not unsettle hegemonies intentionally, making art concerning topics other than ones' pain and struggles can be considered an expression of creative agency. Such practices are not reactive; instead they take initiative and create something new (Björkdahl and Selimovic, 2015).

Second, most participants talked about their art practices in terms of how working on art gives life meaning and makes it “bearable”.

Yeah, because I think life is fucking shitty. And I ask myself a lot of times, like, why do I have to accept this shit? Like, why do I have […] to accept that I see my sisters being beat up and killed for being who they are? Like, why do I have to accept myself getting, like, living through all this violence and shit? […] But when I am writing, when I am doing something, it just makes everything worth it. It just makes everything have a reason. (Shif)

Third, and in relation to such meaning-making properties, participants explained the potential of art in allowing them the freedom to use other kinds of expression than (conventional) language. This creates possibilities to express oneself and to counter experiences of being silenced. We interpret this potential of art practices as enhancing feelings of independence.

Mundane artistic expressions, as well as the meaning-making and counter-silencing potential of art practices, can be considered expressions of agency on the micro level. However, some expressions of agency reach outside the personal realm to different communities, which bring us to the fourth form of agency. Most participants considered their art practices as a way of increasing the (very limited) representation of queer people in general and queer refugees in particular in public spaces.

And people would know about it [their writings] and people would feel connected to it. And because I wrote it in, like, a Moroccan dialect, in Darija, and people—it was like the first time they see someone writes in their own dialect about these kinds of things. So they felt some kind of connection with a person that they didn't know about. (Ghano)

Some participants also described the inspiring and educational properties of art as a relational form of agency that stimulates reflection through increased representation.

Finally, in several instances, participants perceived their art as activism, a fifth way in which participants express agency through their art. While the above expressions can be considered hybrid forms of discursive and creative agency through which dominant hegemonies are challenged both intentionally and unintentionally, those that consider their works more explicitly activist have a clear intention of unsettling dominant norms. Some create political performances to create awareness about societal inequalities at the meso and macro levels. One participant, for instance, uses deejaying to decolonize spaces, to remove western dominant influences.

My, my art, my music, my painting, my writing is my way of activism, is my way of fighting. And I am actually painting to heal or to be therapeutic as a way of resistance against everything that is going wrong in my life and with the system and also with the music. I've already told you, like decolonizing the spaces, bringing back cultures and creating community building. And with the writing is the same. It's vocalizing. The things, the struggles, sharing and connecting. To experience it. (Participant)

In sum, participants challenge and unsettle hegemonies through their art in various ways. While some ways are reactional, intentional or explicitly activist, others might be less intentionally aimed at unsettling hegemonies but they nonetheless take initiative, create something, and therefore still show transformative potential.

### Queer Refugees' Reflexivity

The section above showed how queerness, collectivity and art practices all play an important role in how artistic queer refugees express agency that can potentially unsettle hegemonies and the paradoxes that result from such paradoxes.

However, these different ways of expressing agency are not independent from each other. In fact, queerness, collectivity and art practices are all interwoven and revolve around reflexivity, or “people's ability to reflect on, and understand themselves” (Monro, 2005, p. 18).

I think for most writers, first-time writers, you realize that you also get agency on shaping what the story actually is as you are writing. You make choices about what is important, what is not important. You make choices about the correct order, and you get agency in realizing “oh and this is what that meant.” […] I hate it. But I also love it. And I think that the thing it does for me is that it brings a certain depth to my life, but also a very deep social, emotional, cultural, political depth that I'm very grateful for. And that keeps bringing me back to the writing table. Because it's addictive, it's addictive to be deep. To think deep. To put things at risk, to doubt deeply, to hope deeply. (Osa) [Translated from Dutch].

To understand reflexivity as a mechanism, we propose viewing it through Beech (2011) lens of identity construction within liminality. We thus suggest that being queer, engaging in community and practicing art are all forms of reflexive identity construction. The previously mentioned performance concerning one participant's queer Muslim identity (see section Paradox of Belonging) is an act of knowing and understanding oneself while concomitantly presenting and explaining oneself to an audience. This performance is a strong act of agency involving individual self-reflection in the context of identity construction and the assumption that the combination of queerness, feminism and Islam is highly contradictory. Through their art practice, the participant negotiates these assumed paradoxes, which enables the participant to integrate them into a personalized identity. Moreover, the performance encourages plural and public reflexivity (Turner, 1979).

These reflexive identity constructions seem to be crucial and inevitable. They seem inevitable because it seems highly unlikely, or rather impossible, to not reflect on and construct one's identity within the various paradoxes (as perceived by society) that participants contend with. Thus, participants must position and explain themselves, especially when an identity is perceived as paradoxical or ambiguous. Furthermore, we suggest that reflecting on one's position in society and becoming aware of exclusionary hegemonies as an internal process may lead to external action (activism). Thus, altogether, such acts of identity construction challenge and unsettle hegemonies on all societal levels (micro, meso and macro).

However, we would like to comment on one downside, dare we say, paradox, of reflexivity. Until this point, we have shown that reflecting on one's life or works in society results in a higher sense of agency and power. However, our research has shown that becoming more aware of inequalities and injustices can also make life harder. Most participants expressed being initially naïve about pride celebrations for instance.

It was my very first pride, […] it was a life changing experience. To see all the taboos, that… You think they were wrong, are out in the open! And everybody is expressing themselves and other people are rooting for them. […] I didn't know it's like a capitalism thing. And people go drink and the hetero is taking the piss out of gays. […] But at that time, for naïve, I mean 22 years old me, it was a big deal. (Aram)

For most, pride was once a liberating and joyful experience. However, after realizing the subtle forms of exclusion, particularly due to homonormativity, participants would now rather protest against it. Moreover, in migrating to the Netherlands, most participants brought with them hopes of being free to express themselves. However, it turned out to be far more challenging than they expected due to the subtle nature of hegemonies.

I also experience a lot of shit here with some people. Coming from a south country where you always hear about these great stories, and conditioned to just admire the European experience. And then you come to a place where you see a lot of things actually don't match what you thought it would be. (Shif)

Some participants, thus, said that they no longer maintained long-term goals because the feelings of disappointment were becoming harder to deal with.

I stopped doing the big goals, because when something shifts I get lost and I get so down that I just don't want to do it anymore. […] Because when I was in Morocco, I had this huge goal, big goal. That along with the community I wanted to decriminalize homosexuality, which is ridiculous to think about because we needed more to work on community building. (Ghano)

Becoming more reflective about inequalities and injustices, thus, might cause participants to lose their non-reflective pleasure in hegemonic festivities, and become disappointed or frustrated while losing hope for big societal transformations.

In conclusion, participants find that identifying as queer challenges forms of normativity and in turn creates a sense of belonging to the (or a) queer community. This sense of collectivity provides mental, emotional, practical and financial support as well as collective action (activism). Participants also express agency via art practices, which involve sharing works within and outside these communities and contain the potential to negotiate and communicate ideas of queerness. These art practices may be mundane, meaning-making, counter-silencing, representational, inspirational, educational or explicitly activist. Furthermore, engaging in community, expressing queerness and practicing art all revolve around, and feed, reflexivity. Reflexivity is important in participants' identity construction, in organizing created spaces and in activism. Finally, reflexivity is paradoxical in itself. The more reflective one is the more one may be burdened by the awareness of subtle forms of exclusion and inequality.

## Conclusion and Discussion

In this article, we answer the question of whether and how art-practicing queer refugees can unsettle hegemonies. The answer to our first research question—*Can* art-practicing queer refugees unsettle hegemonies?—is simple. As this paper has illustrated in a very elaborate manner, the answer is “yes, they can”. *How* queer refugees can unsettle hegemonies needs more elaboration about Dutch hegemonies regarding queer refugees and about how art-practicing queer refugees resist such hegemonies at micro, meso and macro levels.

### Dutch Hegemonies Regarding Queer Refugees and How These Affect Queer Refugees

The hegemonies we identified operate on macro, meso and micro levels via discourses, institutional dominance and interpersonal experiences of violence, respectively. In line with earlier studies, those hegemonies are aimed at (1) expecting queer refugees to assimilate into the “Dutch national culture of perceived tolerance and advanced women's and gay emancipation” and (2) differentiating queer refugees as (nearly) incompatible with Dutch culture (Slootman and Duyvendak, 2016). Due to these hegemonies of assimilation and hierarchical differentiation, queer refugees are “caught” in several paradoxes.

One paradox involves representation. Participants see themselves as underrepresented in the media and society at large. What media attention occurs is either one-sided and polarized (i.e., represented as either very good or very bad, but little in-between) or tokenized (highly visible with ongoing pressure to perform perfectly) (Ghorashi and Sabelis, 2013).

Another paradoxical affect concerns the commodification of participants, particularly on gay dating apps. We suggest that this commodification can be explained in light of the politics of homonormativity (Duggan, 2012) in combination with Rubin (1984/1999) concept of sex hierarchy. We propose that native Dutch people may perceive themselves superior in Rubin (1984/1999) hierarchy to the ethnic other. Further, considering that transsexuals and sex workers are perceived as being on the lowest ranks of the sex hierarchy (Rubin, 1984/1999), notions that it is appropriate, as a “consumer,” to objectify and commodify the other may be reinforced. This mode of othering results in the ethnic other being fetishized (objectified and commodified), while simultaneously being feared (rejected).

A further example of how hegemonies affect queer refugees concerns the intersection of various characteristics of participants' identity making it harder to belong in any particular community. Refugees' queerness, as a form of resisting heteronormativity, homonormativity (Duggan, 2012; Vijlbrief et al., 2020), homonationalism and homonostalgia (Mepschen et al., 2010), is considered neither properly Muslim, nor properly Black, nor properly native White Dutch, nor properly gay, according dominant hegemonic discourses.

The final paradox of participation concerns queer refugees being expected (or even pressured) to participate in society, while the manner of participation and representation is determined by the often contradictory expectations, requirements or paradigms of the host society's institutions. Hence, queer refugees are considered neither neutral enough nor other enough. Moreover, participants claim they are often only asked, and remunerated, to work on topics specific to their SOGI, ethnicity and migration story, to share their experiences of pain or loss or to perform the “good example of otherness” (El-Tayeb, 2012). Paradoxically and simultaneously, critics and audiences often wish to experience works by queer refugees “beyond their assumed topics” (i.e., works that are uncritical of issues concerning SOGI, ethnicity or migration background, hence, more neutral).

### Art-Practicing Queer Refugees Unsettling Dutch Hegemonies on Micro, Meso and Macro Levels

We argue that the very existence of queer refugees unsettles Dutch hegemonies. The societally perceived paradoxical identifications (e.g., one cannot be queer and feminist and Muslim at the same time) are challenged by exposing that this societal perception is false. The silencing and invisibility of queer refugees is thus arguably the biggest challenge to overcome. This silencing is countered by constructing and expressing one's identity (via queerness, collectivity and art practices in liminality) and establishing a strong sense of self and community, making it possible to counter such silencing and thus resist and unsettle hegemonies.

We also argue that liminality can be found in (1) queerness, due to notions of betweenness in gender and sexuality (Monro, 2005); (2) forced migration, due to the transitory character of migration and asylum procedures (Ghorashi and Sabelis, 2013; Manjikian, 2014) and (3) art practices, due to the reflexive processes and betweenness of the creation process (Turner, 1979). We propose that this reflexivity can be both discursive—intentional and reactive to hegemonies (Butler, 1999)—and creative—playful and unintentionally unsettling hegemonies (Björkdahl and Selimovic, 2015).

On the micro level, being queer, engaging in community and practicing art are all forms of reflexive identity (re)construction (Beech, 2011). Sharing one's art practices with others can be considered a form of individual self-reflection and identity construction in which one—via art practices—discursively negotiates perceived paradoxical identity markers with specific audiences and integrates these into a personalized identity. Art can be about mundane topics but still be considered expressions of creative agency (Björkdahl and Selimovic, 2015). Such expressions are not reactive, but they still resist societal expectations regarding appropriate topics for queer refugees (i.e., pain, struggle and trauma). Furthermore, there are meaning-making and counter-silencing properties of art practices that, in our view, can be considered as both discursive (Butler, 1997) and creative agency (Björkdahl and Selimovic, 2015).

This brings us to the meso potential of unsettling hegemonies. We found that practicing queerness establishes a notion of belonging to the queer (refugee, migrant, artist) community, which unsettles the paradox of belonging. This sense of community creates mental, emotional, practical and financial support and unsettles western norms concerning individuality. Moreover, within the community, participants do not have the burden of having to explain oneself to others. Community then offers further encouragement for identity construction to be negotiated in a relatively safe way. Furthermore, because they have less fear of being misunderstood within these communities, participants feel safe to share art practices, further reconstructing identities and ideas of queerness. Turner (1979) argues that arts are a form of “plural reflexivity,” affecting and informing the viewer (p. 465). In the context of the participants in this research, we interpret plural reflexivity as sharing, negotiating and communicating one's experiences via art practices, creating representational, inspirational and educational properties for members within and outside their communities. These representations are more nuanced, layered, multi-faceted and realistic, and therefore challenge the paradox of representation. Hence, engaging in community, expressing queerness and practicing art are all reflexive, and playful, elements in identity construction, for both individual and group identities.

On a macro level, community creates the opportunity for collective action (activism) that can trigger public reflexivity (Turner, 1979). Most participants regard their art, and their queerness, as activism. Such activism challenges the paradox of participation because participants create and demand their own terms of societal engagement, as opposed to participating in society in ways they are expected to. We found that participants challenge and unsettle several hegemonic norms (i.e., gender norms, relationship norms, sexual norms, religious norms, societal participation norms, norms regarding individuality, and norms of what is considered art). Through their art, they make political activist performances with the direct intention of creating awareness about societal inequalities. Through their queerness, participants (collectively) demonstrate against different forms of exclusion or injustices. Both performative art and demonstrations address issues such as hegemonic gender norms, homonormativity, racism, islamophobia and sexism. We suggest that these are forms of public liminality that can counter hegemonies by publicly suggesting new social propositions (Turner, 1979).

Art practices thus have the potential to entice or provoke individual, plural and public modes of reflection within and outside the community (Turner, 1979). Engaging in community, negotiating ideas on queerness and practicing art reinforce and encourage reflexivity and playfulness. Queerness, collectivity and art practices offer individual opportunities, as well as communal, or even societal, opportunities to reflect and unsettle hegemonies. We therefore consider reflexivity as a central mechanism in each of these three practices. Further, reflexivity serves as a starting point to concrete actions such as organizing created spaces or activism. We suggest that agency can be both discursive (intentional) and creative (less intentional). Thus, despite the challenges, their use of reflection and playfulness (in liminality) results in queer refugees acting upon their own situations and resisting hegemonic power by unsettling normalized ways of othering.

We thus suggest that queerness (forced), migration and art practices withstand and challenge Dutch hegemonies by enabling a liminal space in which queer refugees' societally perceived paradoxical identities can be reconstructed (Beech, 2011) as multilayered, personalized and unconventional identities, as opposed to assimilated, normative ones. Turning then toward Butler (1999) concept in which identity is argued as performance, we propose the following: when trying to establish a more inclusive society, the potential of personalized, unconventional and multilayered interpretations of societal roles that unsettle hegemonies should be acknowledged.

### Limitations of This Study and Future Ambitions

One of the limitations is that we, the researchers, designed and initiated this project. Currently, we are continuing the project and are doing so in collaboration with the artist-participants. Another limitation has been the contrast between intensive collaboration and the limited possibilities, in terms of time and space, to continue in a same intensive manner. Lastly, by focusing on participants' art practices and making them part of the research, we could use academic resources to make participants' art and struggles more visible. This could, however, be done more durable and consistent; creating and claiming space within academia to co-create, co-curate and co-produce knowledge and art by situating it within sustainable platforms that connect academia and society, which is a continuous challenge and aim of our research team.

## Data Availability Statement

The datasets presented in this article are not readily available because it will not be suitable for reuse by others. The data set is very context specific and it will take someone with the same amount of experience, connections and insights in the specificity of this methodology to understand its layered meanings. Also the research is partly about a group in a vulnerable stage of their lives (refugees). Even if the datasets are pseudomized, refugees often do not want their narratives to be shared in broad circles. Based on our earlier experiences it is crucial to keep the access to the data based on the narratives of refugees in the closed circle of researchers involved in this project. Also, ethnographic data is collected with informal and formal consent from the participants for the specific project in which they are involved. Giving access to the data to other researchers would violate the informed consent given by the participant often based on built trust by the researchers involved in a particular project (we also promised participants in written form that we will not share data with people outside our research team). Furthermore, replication of ethnographic and interpretive qualitative research is virtually impossible, since these data are very context sensitive and based on the situational interaction between the researchers and the participants in the research. In other words, those data make sense in that specific context with those individuals involved. Requests to access the datasets should be directed to f.y.holle@vu.nl.

## Ethics Statement

The studies involving human participants were reviewed and approved by VU Amsterdam Research Ethics Review Committee (RERC). Committee members: René Bekkers (Sociology, until 1-1-2021): chair Ivar Vermeulen (Communication Science, until 1-1-2022): vice-chair Romy van der Lee (Organization Sciences, until 1-2-2023. On leave until April 2021: during this time, researchers can contact Maria Dijkstra) Frédérique Six (Public Administration and Political Science, until 1-12-2023) Peter Versteeg (Anthropology, until 1-1-2024) Contact Koen Leuveld RERC secretary E rerc.fsw@vu.nl T +31 20 59 84034 (Mondays, Tuesday, Thursday and Fridays). The patients/participants provided their written informed consent to participate in this study.

## Author Contributions

FH has collected the data of this project which was analyzed in collaboration with MR and HG (the supervisors of the project). FH has also made the first draft of the present manuscript based on the data which served as a first step toward several rounds of joint writings with the co-authors.

## Conflict of Interest

The authors declare that the research was conducted in the absence of any commercial or financial relationships that could be construed as a potential conflict of interest.

## Footnotes

^1^The artistic works can be found here: https://engagedscholarshipnarrativesofchange.org/scholarship-for-change/art-for-change.
